# The Intrinsic Autonomic Nervous System in Atrial Fibrillation: A Review

**DOI:** 10.5402/2012/490674

**Published:** 2012-06-19

**Authors:** Bo He, Benjamin J. Scherlag, Hiroshi Nakagawa, Ralph Lazzara, Sunny S. Po

**Affiliations:** ^1^Department of Cardiology, Renmin Hospital of Wuhan University, 238 Jiefang Road, Wuchang, Wuhan, Hubei 430060, China; ^2^University of Oklahoma Health Sciences Center, Heart Rhythm Institute, 1200 Everett Drive, Room ET6E103, Oklahoma City, OK 73104, USA

## Abstract

The procedure of catheter ablation for the treatment of drug resistant atrial fibrillation (AF) has evolved but still relies on lesion sets intended to isolate areas of focal firing, mainly the myocardial sleeves of the pulmonary veins (PVs), from the rest of the atria. However the success rates for this procedure have varied inversely with the type of AF. At best success rates have been 20 to 30% below that of other catheter ablation procedures for Wolff-Parkinson-White syndrome, atrioventricular junctional re-entrant tachycardia and atrial flutter. Basic and clinical evidence has emerged suggesting a critical role of the ganglionated plexi (GP) at the PV-atrial junctions in the initiation and maintenance of the focal form of AF. At present the highest success rates have been obtained with the combination of PV isolation and GP ablation both as catheter ablation or minimally invasive surgical procedures. Various lines of evidence from earlier and more recent reports provide that both neurally based and myocardially based forms of AF can separately dominate or coexist within the context of atrial remodeling. Future studies are focusing on non-pharmacological, non-ablative approaches for the prevention and treatment of AF in order to avoid the substantive complications of both these regimens.

## 1. Historical Background: From Bedside to Bench

The examination of a patient with chest palpitations and an irregular and rapid pulse was more definitively diagnosed and designated as auricular fibrillation with the advent of the electrocardiogram at the beginning of the 20th century. This clinical observation engendered an ongoing polemic for many subsequent decades regarding the mechanism underlying this most common disordered cardiac rhythm. Essentially two schools of thought developed, each with its chief proponents, each based on accumulated experimental evidence, which were apparently contradictory to one another. Specifically, Scherf and his associates [1, 2] promulgated the focal theory of atrial fibrillation (AF) by demonstrating that substances, such as aconitine [1] or acetylcholine applied to the atrial appendage or to the area of the sinus/AV node, [2] could induce a rapid auricular tachycardia or auricular fibrillation, respectively. Isolation of the appendage or local cooling was consistently able to suppress the tachyarrhythmia thereby promoting the conclusion that AF was focal. On the other hand, initial studies by Moe [3] provided strong evidence for multiple wavelets (reentrant circuits) occupying the atria during AF induced by triggering atrial premature beats along with vagal nerve stimulation. More sophisticated mapping techniques employed by Allessie and coworkers [4], using the same experimental model, clearly demonstrated the existence of several reentrant circuits traversing the atria giving unambiguous support for Moe's multiple wavelet hypothesis.

## 2. Evolution of Catheter Ablation for Clinical Forms of AF: The Myocardial Hypothesis

The initial attempts at catheter ablation for AF, in patients resistant to drugs and cardioversion, were based on the rationale that the underlying mechanism for AF was due to multiple reentrant wavelets encircling the atria. Therefore, transmural lesions sets, similar to those produced surgically, were instituted using radiofrequency energy delivered to the left and/or right atrial endocardium [5, 6]. In part, this approach was an extension of what was found to be highly successful in cutting the reentrant circuits involved in Wolff-Parkinson-White (WPW) syndrome, atrioventricular junctional reentrant tachycardia (AVJRT), and atrial flutter (AFL). These attempts to follow the surgical findings of Cox and associates [7] were modified as a result of the surprising and seminal findings by Jaïs et al. [8] and Haïssaguerre et al. [9] which revealed that *focal *firing arising from the pulmonary veins (PVs) were the apparent initiators of AF. Other investigators found additional focal firing sites in the myocardial sleeves of the superior vena cava [10] and the myocardium within the ligament of Marshall [11]. Of particular interest, this focal form of AF was singularly resistant to standard antiarrhythmic drugs and cardioversion. In contrast, the AFFIRM trial [12], while focusing on the dichotomy between rhythm and rate control, clearly showed that a substantial portion of the unselected AF population was responsive to antiarrhythmic drug therapy, whereas drugs were not effective in converting approximately half of the treated population. It is reasonable to assume that this latter cohort consisted of candidates for catheter ablation.

Undeterred by these apparent differences in the AF population, the clinical electrophysiologist focused their catheter ablation techniques on those patients with PV and non-PV focal firing. However, ablation of focal firing sites within the PVs was quickly abandoned since PV stenosis became a potential serious complication for this approach. Most centers subsequently adopted the application of multiple linear radiofrequency applications well outside the PV ostia to provide a circumferential barrier between the focal PV source of firing and the atria [13–15] thereby avoiding the possibility of PV stenosis. Although the results of this approach were relatively positive (70–80% success for paroxysmal AF), it was noted that (1) there was “…no significant relationship between lesion completeness and clinical outcome…”[13]. Others found that PV isolation was “not crucial in determining clinical success” [16, 17]. (2) PV firing, in the great majority of cases, unexpectedly stopped after PV isolation [18]. These findings have led to the conclusion that some influence coming from the atria must be a critical factor in maintaining PV firing. As if to provide the missing factor(s), Nademanee et al. [19] reported that ablation of complex fractionated atrial electrograms (CFAE) during AF could be performed, thereby increasing the success rate to >90%. Although this procedure avoided PV isolation, as few as 40 and as many as 140 radiofrequency applications were performed in these cases [19]. However, Oral et al. [20] performed ablation of CFAE in patients with chronic AF and did not successfully reproduce the results as Nademanee et al. reported. Whether the presence of CFAE is culpable or innocent also remains difficult to determine even though ablation of CFAE has been widely adopted. At present, the catheter ablation procedure consists of at least 4 steps with the application radiofrequency energy attempting to induce transmural myocardial lesions: (1) Circumferential lesion sets to isolate both left and right PVs, (2) A linear lesion connecting the circumferential lesion sets, (3) A linear lesion between a circumferential line and the mitral annulus, (4) Ablation of CFAE [21]. It should be pointed out that lesion sets 2 and 3 were made to preempt an iatrogenic macro-reentrant arrhythmia resulting from lesions sets, 1, that were performed to isolate the PVs.

## 3. Catheter Ablation for AF: Clinical Outcomes

Mainly using PV isolation with additional lesion sets mentioned above, a world-wide survey of catheter ablation for AF [22] from 182 centers reported on 20,825 procedures on 16,309 patients between the years 2003–2006. “Of the 16,309 patients with full disclosure of outcome data, 10,488 (median 70.0%) became asymptomatic without antiarrhythmic drugs (AADs) and another 2,047 (10.0%) became asymptomatic in the presence of previously ineffective AADs over 18 months (range, 3–24) of follow-up. Success rates without AADs and overall success rates were significantly larger in 9,590 patients with paroxysmal AF (74.9%) than in 2,800 patients with persistent AF (64.8%) and 1,108 patients with long-lasting AF (63.1%) (*P* < 0.0001). Major complications were reported in 741 patients (4.5%).” Interestingly, these findings do not show any significant differences in success rates reported in studies from individual centers nor from earlier consensus statements [23]. More recent long-term studies of the outcomes for catheter ablation for AF have been even more disappointing. The long-term success of a single catheter ablation procedure for AF with a follow-up period of five [24] or six [25] years has ranged from 29% to 55%. Ablation of the autonomic neural elements on the heart, either as a standalone or adjunctive procedures with PV isolation have been promulgated with varying degrees of success (see below).

## 4. The Autonomic Nervous System and Focal AF: The Neural Hypothesis

The relationship between the autonomic nervous system's cardiac innervation from the brain and AF was well established during the last century. Stimulation of the vagosympathetic trunks to heterogeneously shorten refractoriness across the atria (dispersion of refractoriness) and the introduction of a premature or series of atrial premature beats to induce and sustain AF became the accepted mode for basic studies of AF [26, 27]. However, toward the end of the 20th century, new findings began to emerge. Specifically, another aspect of the autonomic innervation of the heart, encompassed by the ganglionated plexi (GP) on the heart itself and also on some of the large vessels close to the heart [28–30] gave rise to the concept of an intrinsic cardiac autonomic nervous system. In essence, there is the autonomic innervation to the heart from the brain and the spinal cord (extrinsic system) and the ganglia plexi on the heart itself comprising the local autonomic nervous systems (intrinsic system). This intrinsic system, with each of the major GP situated at the PV-atrial entrances, can contain as few as 200 to as many as 1000 neurons [31, 32]. The intrinsic cardiac autonomic nervous system (ICANS) on the heart and within the pericardium, serves as more than a relay station for the extrinsic projections of the vagosympathetic system from the brain and spinal cord to the heart. It functions as an integrative system which acts cooperatively with the extrinsic innervations but can act independently to modulate numerous cardiac functions, for example, automaticity, contractility, conduction, and so forth [30].

Within the past decades a series of studies from our laboratories and others have related the stimulation of the ICANS, either electrically [33, 34] or chemically, with autonomic neurotransmitters, [35–38] to induce focal firing arising from PV and non-PV sites closely simulating the focal firing described clinically. Moreover, ablation of the major GP at the PV-atrial entrances either eliminated or markedly diminished AF inducibility [39–42]. Also, the administration of autonomic blocking agents has been shown to suppress triggered firing in the PV myocardium *in vitro* [43] and can inhibit ICANS-based AF *in vivo* [44]. Another important aspect of focal AF were the studies showing that this form of AF was not responsive to standard antiarrhythmic drugs, for example, Class III or IC agents [45, 46]. These findings are in direct contrast to the series of studies by The Netherland investigator whose rapid pacing model of AF was readily controlled by various classes of antiarrhythmics [47].

Mapping studies of the long-term atrial pacing model to induce sustained AF found that macro- and multiple reentrant circuits were the responsible mechanism for this form of AF [48]. The apparent discrepancy may readily be explained by the presence of coexisting focal and macro- or multiple reentrant forms of AF [46] in which one or the other dominates or controls the arrhythmia. For example, Chou et al. [45] found that ibutilide was ineffective in terminating the focal discharges, that is, “nonreentrant mechanism” in the PV during sustained AF. Niu et al. [46] reported that propafenone was ineffective in terminating PV firing induced AF. However, the same dose of propafenone readily converted AF to sinus rhythm when GP ablations markedly reduced CFAE and pacing could capture the residual macroreentrant circuit that became the predominant mechanism maintaining AF. This difference in drug responsiveness was consistently demonstrated in an experimental model in which the same heart could show both forms of AF or circumstances where both mechanisms appeared to be operating simultaneously [46]. It is interesting to note that in 3 patients whose AF continued after multiple CFAE ablations, ibutilide converted AF to sinus rhythm as reported by Nademanee et al. [19]. The importance of the coexistence of the focal (drug resistant) and drug responsive forms of AF will be elaborated below.

## 5. Alternative Methods for Catheter Ablation of AF: Targeting the Ganglionated Plexi

The earliest study of selective GP ablation was reported by Platt et al. [49] who described the identification of the GP at the PV-atrial junctions by applying high-frequency stimuli to these nerve clusters. In patients with persistent forms of AF, the response was a marked slowing of the ventricular rate (≥50%) during AF. Ablation of these GP terminated the persistent AF in 23/26 patients who had a complete study with an overall success rate of 96% during a short 6-month follow-up. More recent studies have reported highly variable success rates ranging from 25% to 78% after ≥1 year of follow-up [50–53]. It should be pointed out that in some of these studies GP ablation was performed by on anatomic identification of GP sites. No high-frequency electrical stimulation was used to determine that they were ablated after radiofrequency applications [51, 52]. Furthermore, the study showing the lowest success rate may have only performed partial ablation by missing the largest GP situated anteriorly between the right superior and inferior PVs, the anterior right GP. In addition, in this study [50], the GP were approached epicardially, the anterior right GP which is situated closely adjacent to the phrenic nerve precluded the separation of GP and phrenic nerve by stimulation and, therefore, that particular GP was not ablated [54]. Experimental studies have shown that partial GP ablation is not only less effective than more complete GP ablation but partial ablation of the GP may increase the incidence of AF by exacerbating the heterogeneity of refractoriness across the atria thereby promoting macro-reentrant AF [55, 56].

## 6. Combined Methods for Catheter Ablation of AF: GP Ablation and PV Isolation

The first clinical study showing the relatively long-term success of a combination of GP ablation and PV isolation was reported by Pappone et al. [57]. In a nonrandomized study of 297 patients with paroxysmal AF, undergoing left atrial circumferential ablation to isolate the pulmonary veins, these investigators found that some 34% showed marked slowing of the ventricular response along with hypotension during the application of radiofrequency energy to 4 specific areas adjacent to the PVs. In a 12-month follow-up, those 101 patients showed 99% freedom from AF. In a series of 83 patients with paroxysmal and persistent AF, Nakagawa et al. [58] provided similar results in that the freedom from symptomatic AF was 95% and no AF or AT at 22 months was 86% after a single procedure targeting both GP and performing an antral type PV isolation. Similar results were also confirmed by a recent randomized study [59] showing that addition of anatomic GP modification to PV isolation confers significantly better outcomes than PV isolation alone during a follow-up period of 12 months.

Up to this time, the major source of results in which both GP ablation and PV isolation have been performed as a single procedure has been via minimally invasive surgical techniques. A survey was conducted of 5 such studies from independent surgical investigators [60–64], showed success rates ranging from 83 to 93% with follow-up periods spanning 6 to 13 months. Most of these patients had persistent or long standing persistent AF. Patients with paroxysmal AF were in the minority.

## 7. Coexistence of the Myocardial and Neural Mechanisms in the Clinical Forms of AF

Taking into account what we have learned from previous basic studies and from more than a decade of clinical experience with catheter and surgical ablation of AF, perhaps we can construct a scenario which incorporates both the autonomic and myocardial mechanisms in an attempt to explain the initiation as well as the maintenance of AF in patients. As far as initiation of AF, it should be noted that the myocardial tissues, that is, PVs, superior vena cava and “Marshall Bundles,” comprising the major sources of focal firing derive from a common phylogenetic and embryological origin, namely, the sinus venosus [65]. Functionally, they show a greater sensitivity to the cholinergic and adrenergic neurotransmitters than adjacent atrial tissues [43]. The basic and clinical evidence gathered over the past decade would support the hypothesis that the focal firing arising from PV and non-PV sites are neurally based and due to a hyperactive state of the ICANS. A fundamental question is Why do these tissues become hyperactive in some of the population, in particular, disproportionately in persons from 60–80 year old? An early report by Kaijser and Sachs [66] studied cardiovascular responses to autonomic influences in healthy women and men comprising groups, age 25, 45, and those between ages 60–80. “There seems to be only a moderate attenuation of autonomic cardiovascular responses to about 60 years, after which there is a more rapid decline.” In this regard, Smith et al. [67] tested the function of the ICANS, weeks after denervation, which is separation of the extrinsic from the intrinsic autonomic systems. Not only did the intrinsic GP neurons remain viable but their responsiveness was enhanced. A more recent study by Zhang et al. [68] showed that vagosympathetic trunk stimulation could abolish neurally firing within the anterior right GP closely associated with the adjacent right superior PV. As mentioned above, both electrical and chemical stimulation, that is, induced hyperactivity, of these GP, which in turn, triggered focal firing of adjacent PVs leading to AF. The implications of these separate findings suggest that with age extrinsic control of intrinsic activity is reduced allowing not only independent operation of the GP at the PV atrial junctions but also “muscarinic facilitation of orthodromic neuronal activation increased” [67]. In other words, the attenuation of extrinsic control with age predisposes the elderly to paroxysmal AF which in time progresses to more persistent forms.

 Based on these findings, we hypothesized that extrinsic control of the intrinsic autonomic nervous system leads to hyperactivity of the GP which in turn could provide the increased propensity for atrial fibrillation. To test this hypothesis, we performed a chronic study in which the nexus point between the extrinsic and intrinsic autonomic nervous systems was ablated in dogs (*n* = 5) and the development of AF monitored from an implanted pacemaker. Over a period of 10 weeks, there was a progressive increase in the AF burden starting at 4-5 weeks and increasing thereafter. No AF burden was recorded in the sham operated (*n* = 5) group [69].

 Elegant basic studies promoted the hypothesis that “AF begets AF” [70]. The electrophysiological changes, namely, atrial refractoriness and pacing maladaptation, as well as anatomic changes [71] implicitly showed that AF could progress from the paroxysmal or the episodic form to the persistent form. More recently, it has been shown that AF also begets autonomic remodeling [42] which can significantly add to functional and myocardial remodeling, all of which promote the AF substrate. Specifically, as AF burden increases, GP activity increases resulting in excessive release of muscarinic and adrenergic neurotransmitters locally and via their axonal fields to smaller clusters of ganglia which comprise the interconnected neuronal network covering the atria [30–32, 72]. As the increased neural activation spreads to peripheral atrial sites, the excessive neurotransmitter release locally engenders another exacerbating factor for the “AF begets AF” effect. Recent studies in the dog heart with sustained AF have demonstrated that acetylcholine applied locally to the atria in increasing concentrations can cause site-specific intermittent, then continuous CFAE [73]. Moreover, the spread of rotor-like firing and CFAE occurs from the GP at the PV-atrial junctions to the PV in one direction and toward the atrial appendage in the other direction [74]. In extreme cases, this “metastasizing” of the CFAE can envelope the atrial appendage providing triggers capable of initiating AF from these secondary sites [38].

 In patients with long-standing persistent AF presenting with extensive CFAE recordings throughout the left atrial appendage, attempts at isolation resulted in a “dismal” success rate without total electrical ablation of the atrial appendage [75]. It would appear that CFAE is to the abnormal atrium what focal firing is to the abnormal PV. This may help to explain some unusual findings reported clinically. For example, the cessation of PV firing [18] and/or termination of AF after complete or even incomplete PV isolation [16, 17]. In these cases, the linear ablation line may also pass through and ablate the GP at the PV-atrial antra [57] thereby reducing the efferent neural input, that is, parasympathetic and sympathetic to a level that would not sustain paroxysmal AF [57]; or, the PV isolating lesions may also interrupt CFAE serving as additional focal firing sources sustaining both paroxysmal and persistent AF [58].

Another, seemingly paradoxical, clinical finding is exemplified by the report by Danik et al. [76] on a series of 18 patients whose AF duration ranged from 1–12 years despite various drug regimens. These investigators were able to induce AF with rapid atrial stimulation after acute GP ablation in 17 of 18 patients. Subsequently, PV isolation was performed in the 17 patients who then were put on antiarrhythmic drugs for 6 weeks, with 3 remaining on drugs during a mean follow-up of 15 ± 2  months. Freedom from AF recurrence was 94%, at least 20% higher than reported in the two consensus statement listed above [22, 23]. We suggest that after ablation of the GP and marked reduction of PV firing [58] burst atrial pacing can induce macroreentrant circuits in the remodeled atria [58]. During follow-up drugs that were previously ineffective against the focal form of AF [43, 44] now can maintain sinus rhythm. As sinus rhythm persists, there is a reversal of remodeling, “sinus rhythm begets sinus rhythm,” providing long-term freedom from AF recurrence.

 It should be mentioned that GP ablation as an adjunctive procedure to pulmonary vein isolation (PVI) still does not achieve the same degree of success as achieved by catheter ablation procedures targeting patient with WPW, AVJRT and AFL. Obviously AF is a more complex arrhythmia in which more than one mechanism or a coexistence of mechanisms are involved (see above). Also, the ablation of the major GP at the PV-atrial junctions has been shown to achieve success rates as high as 99% [57]. As would be expected the success rates should be reduced as AF “metastasizes” to the atrial neural network resulting in persistent and long-standing-persistent AF. Clinical studies in which PVI plus major GP ablation has been performed in patients with both paroxysmal and persistent AF have reported success rates of 86% [77].

## 8. Future Approaches for the Treatment of the AF Patient

 Despite the successes of catheter ablation for AF, over more that a decade, it has not achieved the same efficacy compared to catheter ablation of WPW, AVNRT, and AFL. Furthermore, the relative complexity, amount of myocardium destroyed and the overall complication rate require much needed improvement. What has been learned from both basic and clinical investigations have begun to suggest different modalities, particularly neural based, that may supplant catheter ablation, for the prevention and treatment of some forms of AF. Our recent studies [78–80] demonstrated that bilateral low level electrical stimulation of the vagosympathetic trunks at a voltage 10% or even 50% below that, which slows the sinus rate or AV conduction, can significantly prevent AF inducibility. In the normal dog heart, subjected to 3 hrs of low level vagal nerve stimulation, there was a progressive and significantly increased AF threshold compared to baseline values. Controls showed no change after the same time period. We also found that bilateral low level vagal nerve stimulation can prevent and reverse atrial remodeling induced by rapid atrial pacing as well as suppress AF-induced by strong cholinergic stimulation [79]. These results were further confirmed by our ongoing studies when stimulating from the right vagosympathetic trunk alone [81] or when we positioned a pacing catheter in the superior vena cava to stimulate the preganglionics of the vagosympathetic trunk to achieve low level vagal nerve stimulation without having to surgically expose the nerves [82]. Evidence from other investigations suggested that these low level voltages release vasoactive peptides and nitric oxide, both of which manifest strong antiadrenergic action [83–85]. Such an effect would suppress triggered firing at PV and non-PV sites. Whether the use of this modality can be made into a feasible clinical tool and in which group of AF patients this would be efficacious remains to be determined. The optimal treatment may lie in the development of a noninvasive method for prevention and termination of AF.
